# Health system, socio-cultural, economic, environmental and individual factors influencing bed net use in the prevention of malaria in pregnancy in two Ghanaian regions

**DOI:** 10.1186/s12936-019-2994-5

**Published:** 2019-11-12

**Authors:** Matilda Aberese-Ako, Pascal Magnussen, Gifty D. Ampofo, Harry Tagbor

**Affiliations:** 1 University of Health and Allied Sciences, PMB 31, Ho, Volta Region Ghana; 2 Faculty of Health and Medical Sciences, Centre for Medical Parasitology, University of Copenhagen, Copenhagen, Denmark

**Keywords:** Ghana, LLINs, Malaria in pregnancy, Health facilities, Health workers, Pregnant women, Socio-cultural, Economic, Environment, Gender power relations

## Abstract

**Background:**

Improving maternal health remains a priority to the Ghanaian government. Consequently, it has implemented the World Health Organization recommendation of distributing free long-lasting insecticidal nets (LLINs) to pregnant women—one of the effective strategies to combating malaria in pregnancy. However, the burden of negative outcomes of malaria in pregnancy such as low birth weight and miscarriages is still high. This may be related to the health system, socio-cultural and economic dynamics that influence LLIN use, but their role is not well understood. This ethnographic study sought to understand health system, socio-cultural, economic and environmental dynamics in utilization of LLINs among pregnant women in two Ghanaian regions.

**Methods:**

An ethnographic study design was used. In-depth interviews and conversations were conducted among health workers, pregnant women and opinion leaders. Observations were conducted in 12 communities and eight health facilities. Ethical clearance was obtained from the University of Health and Allied Sciences’ Research Ethics Committee. Nvivo 11 was used to support data coding. Data were triangulated and analysed using a thematic approach.

**Results:**

Findings suggest health system, socio-cultural, economic, environmental and individual factors influenced LLIN use. Health facility readiness in stocking LLINs influenced ownership and use. Receiving appropriate information from health providers and encouragement from public officials improved LLIN use. Women with a history of LLIN use prior to becoming pregnant and women who had young children remained consistent users. Experiencing irritating effects of LLINs and preference for traditional methods to wade off mosquitoes, reduced LLIN use. Pregnant women whose household and family members used LLINs were influenced positively to use them. Gender power relations between husbands and wives influenced women’s use of LLINs. The type of housing and weather conditions contributed to inconsistent use. Staying out late for business purposes and to converse, exposed pregnant women to mosquito bites.

**Conclusion:**

Giving out LLINs at facility level should be accompanied with comprehensive information, which is relevant to the socio-cultural context that women live in. Mass distribution should factor in individual and public information to promote community acceptance and proper use of ITNs. Facilities should be encouraged to constantly maintain LLINs stock in order to ensure that ANC registrants receive LLINs for use.

## Background

Although malaria is preventable it remains a leading cause of morbidity and death especially in sub-Saharan Africa [1–4], which carries the highest share of the global malaria burden, with 92% of malaria cases and 93% of malaria deaths worldwide in 2017 [3]. The most at risk population include pregnant women and children [1, 5, 6]. Malaria in pregnancy is associated with negative outcomes such as illness requiring hospitalization, anaemia and to the unborn child abortions and still births, especially in first time pregnancies [6, 7]. Constant and appropriate use of long-lasting insecticidal nets (LLINs) during pregnancy is one of the World Health Organization (WHO) recommended measures to control malaria in pregnancy [6, 8, 9], including full coverage with free distribution of LLINs to ensure universal access. This policy has been implemented through the institution of the Global Fund for malaria, HIV and TB, and other interventions in Ghana [10].

However, regardless of all the efforts, knowledge [11], awareness [9] and ownership of LLINs seem not to guarantee appropriate usage of LLINs [12–16]. The study explored how health system, socio-cultural, economic, environmental and individual factors influence ownership and use of LLINs among pregnant women in two Ghanaian regions.

## Methods

### Selection of research area

The study was conducted in five districts, three in the Ashanti and two in the Volta regions of Ghana. Eight health facilities: (five government and three faith-based) and twelve communities were chosen for the study. A three-stage selection process was used: (i) Five districts were selected; (ii) the district hospitals in the five districts automatically qualified to participate in the study; (iii) the study team visited the hospitals and went through antenatal care (ANC) case records. The total number of malaria in pregnancy cases from January, 2015 to March, 2018 in the different communities was recorded and the community with the highest recorded number of malaria in pregnancy cases in each facility was chosen to participate in the study. Information on communities that each facility served was obtained from the offices of the study districts’ health directorate. Most of the communities had an average population of 10,000 inhabitants. However, one of the districts in the Volta region had communities with an average of 2000 inhabitants, so six communities with the highest number of malaria in pregnancy cases were combined to form two study units (Table 1). The study team then conducted community entry activities such as paying courtesy calls on the assembly members and chiefs and holding meetings with a cross section of opinion leaders to inform and to seek their permission to conduct the study in their communities. Interactions and interviews with pregnant women revealed that in some of the chosen communities specific health facilities were preferred for ANC services. Three of such facilities, which are faith-based health facilities comprising of two Christian and one Moslem health facility were included in the study (Table 1).

**Table 1:** Study health facilities and study communities with pseudonyms in the Ashanti and Volta Regions

Region	Type of health facility		Government owned	Mission owned	No of facilities	No. of communities
	Hospital	Health centre				
Ashanti^a^	3	1	2	2	4	4
Volta^b^	2	2	3	1	4	8
Grand total	5	3	5	3	8	12

^a^Study facilities in the Ashanti region have been given the pseudonyms: ASFacility01, ASFacility02, ASFacility03 and ASFacility04. Study communities in the Ashanti region have been given pseudonyms: ASCommunity01, ASCommunity02, ASCommunity03, ASCommunity04

^b^Study facilities in the Volta region have been given the pseudonyms VRFacility01, VRFacility02, VRFacility03, VRFacility04. Study communities in the Volta Region have been given pseudonyms: VRCommunity01, VRCommunity02, VRCommunity03, VRCommunity04

### Study design

This ethnographic study used non-participant observations, transect walks, conversations, in depth interviews (IDIs) and case studies to obtain data from health workers, pregnant women and community members, from April, 2018 to March, 2019. The research team comprised of eight research assistants and each was assigned to a facility and at least one community, to spend long and active periods to learn, experience and represent the lives of study subjects in their natural setting [17–19]. To prevent a Hawthorn effect, observations were conducted intermittently in the eight facilities and 12 communities [20]. MD participated in data collection and also supervised the implementation of the study. The Ewe and Twi languages were used for IDIs with pregnant women and community members in the Volta and Ashanti regions, respectively.

Transect walks were carried out in the study communities to afford the team a visual picture of environmental issues such as mosquito breeding sites, hygiene conditions, type of housing, nearness and access to health facilities and other important areas in the communities.

### Selection of study participants

A research assistant carried out observations in a health facility and interacted with the pregnant women who attended ANC. The study was explained to them and those who were interested were taken through a consenting process and recruited to participate in IDIs, after a written consent had been obtained. Pregnant women were also recruited from the communities, using the snowball method. The first pregnant woman to be identified helped the research assistant to identify others. Opinion leaders such as assembly members, linguists, traditional birth attendants were also invited to participate in IDIs.

Case studies were purposively selected from women who regularly attended ANC on a monthly basis and those who were irregular or skipped their ANC appointments. Pregnant women who were clinically diagnosed with malaria and were willing to participate in the study were included as case studies. They were visited several times at home for observations and conversations with research assistants. Health workers who provided ANC service and had been working in a facility for more than 1 year, majority of whom were midwives, were selected to participate in the study. Follow up conversations and interviews were conducted with procurement officers, laboratory personnel and officials at the district health directorate, to clarify some of the issues raised in IDIs with health providers and health managers. ANC unit managers commonly referred to as in-charge, facility managers such as senior medical officers, physician assistants and administrators, were also interviewed to help understand managerial and administrative issues. Details of the different category of study participants and the methods used for data collection have been presented in Table 2.

**Table 2:** Data collection methods and categories of respondents

Region	Category of respondents	IDIs	Conversations	Case studies	Transect walk
Ashanti^a^	Health Managers	8	4	0	3
	Health Care providers	11	20	0	–
	Pregnant women	30	25	4	–
	Opinion Leaders	10	5	0	–
	Procurement officers	1	2	0	–
	Laboratory officials	0	2	0	–
	DHD officials	0	2	0	–
	Total	60	60	4	3
Volta Region^b^	Health Managers	8	4	0	4
	Health Care providers	12	20	0	–
	Pregnant women	40	32	8	–
	Opinion Leaders	14	6	0	–
	DHD Officials	0	2	0	
	Total	74	64	8	4

^a^Observations were carried out intermittently in 4 health facilities and 4 large communities from May, 2018 to March, 2019 in the Ashanti Region

^b^Observations were carried out in four health facilities, 6 small and 2 large communities from April, 2018 to March, 2019 in the Volta Region

### Data analysis

Interviews were tape recorded and transcribed verbatim to preserve interviewees’ original messages and experiences. Interviews in Ewe and Twi were transcribed into English to enable easy analysis and comparison. Qualitative analysis software, Nvivo Version 11 was used to generate a coding list on common themes that arose from the data (interviews, observation notes and conversations). Two coders, independently coded the data thematically. The analysis aimed at identifying similarities, patterns, differences, and contradictions in the information presented by interviewees [21]. Main themes that were identified from the analysis formed the basis for interpreting and reporting on study findings.

### Ethical issues

Ethical clearance was obtained from the University of Health and Allied Sciences’ Research Ethics Committee [UHAS-REC/A.I Ul 17−18]. Written consent was obtained from all interview participants, while oral consent was obtained from study participants that conversations were held with and for observations. Permission was sought from district directors of health and facility managers. Besides actual country and region names, pseudonyms have been used for individuals and facilities’ names, to protect informants’ identity.

## Results

### How multiple level factors interacted to influence pregnant women’s decision to use LLINs

Multiple level factors such as how restocking of LLINs was organized in facilities and the content of health information on LLINs given to ANC clients influenced LLIN use. Others were contextual factors such as socio-cultural, environmental, economic and individual factors, which intersected and played out in the daily lives of women. The study observed at ANC sessions that in three out of six cases, health providers failed to provide comprehensive information on LLINs to ANC registrants. The reasons included providers taking it for granted that LLINs are common in Ghana, so women automatically knew how to use them and that women knew the reasons why they were being provided LLINs. Sometimes health workers did not seem to have adequate time to provide comprehensive information, due to large numbers of clients and health workers appeared to be more concerned with ensuring that registrants did not go to keep the LLINs or sell them. To prevent such occurrences some facilities tore the LLIN package before issuing it. Information on LLINs was hardly included in the group health talks that were provided in some of the facilities in the mornings, before the onset of ANC services. Nevertheless, when pregnant women were diagnosed of malaria, health providers deciphered that they were not using LLINs or that they were inconsistent users, which was mostly true. They were then given comprehensive information on LLIN use, after they had received treatment. A health provider shared her predicament:

“*It is very unfortunate that most of the pregnant women don’t sleep under the bed net given to them and because of that, today I have recorded two malaria cases. I questioned them [the pregnant women infected with malaria] why they were not sleeping under the bed net and they told me, when they sleep under the bed net they feel hot, so they have stopped sleeping under the bed net…. I advised them to wear protective clothes when they are outside during the night.”* (ASFacility02, IDI, ANC manager)

The campaign on mass distribution of LLINs provided little or no information on LLINs to communities and households. Consequently, community members did not appreciate their relevance and used some of the fairly new LLINs for fencing gardens and livestock pens, as pillows and to dry palm-nut seeds.

**Fig. 1:**
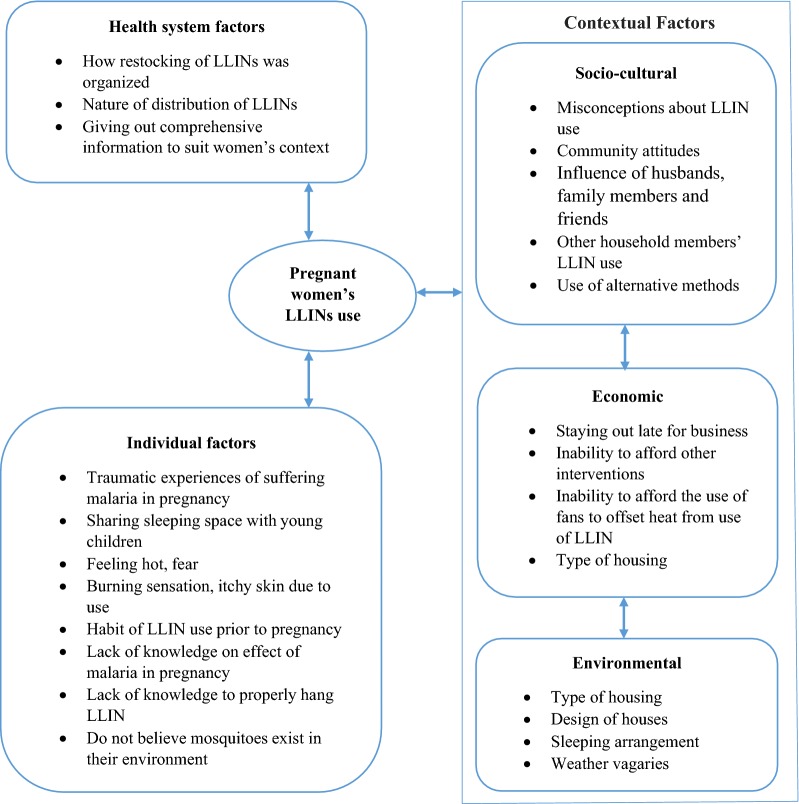
Different connecting factors influencing pregnant women’s LLIN use in study Communities

Pregnant women who received some information from health workers, radio/TV and community and national leaders, overcame community barriers to LLIN use, such as misconceptions about LLIN use, negative community attitudes towards use and weather vagaries (Fig. 1). Traumatic experiences of suffering malaria in pregnancy and the subsequent admonition and comprehensive information provided by health providers and having young children who shared sleeping space with mothers promoted the use of LLINs. Additionally, gender power relations within the household and other household members’ LLINs use influenced women’s use. The nature of housing and sleeping arrangement also influenced LLIN use. Economic status had a mixed effect on LLIN use.

An unexplained occurrence was the high incidence of miscarriages among women who were not using LLINs in one of the districts in the Volta Region, which is the only coastal district of the five districts studied. There appeared to be more mosquitoes in the district, but LLIN use was not different from the other four districts. A detailed discussion of health system, contextual and individual factors are presented in the rest of the results.

### Health system factors

Interviews, observations and conversations with health care providers and pregnant women in the eight health facilities revealed that most ANC registrants were given a free LLIN on the first ANC visit, following confirmation of pregnancy. Eleven of the twelve case studies who were visited at home, owned and used LLINs that were in good condition, only one adolescent had an old LLIN, which had been stitched. The twelfth case study removed her LLIN after delivery, because her room had poor ventilation and was too hot to use an LLIN. Table 3 gives a summary of the number of women who owned one or more LLINs and those who did not own an LLIN in the two regions and the sources of ownership.

**Table 3:** LLIN ownership among pregnant women and source of LLINs acquisition

Region	Ashanti region	Volta region
Number of women interviewed or conversed with	55	72
Number that owned one LLIN	41	63
Number that owned 2 or more LLINs	5	13
Number that did not own an LLIN	14	9
Sources of LLIN acquisition	ANC, Community mass distribution and child welfare clinic	

### Stocking of LLINs at health facilities

All eight facilities in the two regions kept a minimum stock of LLINs and requisitioned for LLINs from the district health directorate (DHD). Unlike drugs and other medical supplies that were requisitioned through the procurement unit, the ANC and the Reproductive and Child Health units (RCH) were allowed to requisition LLINs directly from the DHD.

LLINs were available in the four facilities in the two districts of the Volta region. The facilities only experienced stock outs for short periods of 2 to 4 weeks, during the 1 year period that the facilities were observed. In one of the two districts of the Volta Region, the DHD usually conveyed the LLINs in the DHD’s vehicle to their facilities, whenever a request was made. In the second district, ANC managers of the two study facilities usually requested for a vehicle from their facilities, which was readily released to transport LLINs from the DHD. Arrangements were made for ANC registrants who were not issued LLINs to return at a later date to receive them. The few women who did not own LLINs in the Volta region started ANC at different facilities, where they had not been given LLINs. Health providers argued that the guideline indicated that only a first time registrant of a current pregnancy should receive an LLIN. If a woman transferred from one ANC facility, she was not eligible to receive a LLIN in the second facility, because she was supposed to have received one from her first facility.

The higher number of women not owning LLINs in the Ashanti region compared to the Volta region, was because three of the four facilities in the Ashanti region experienced frequent stock outs. The ANC in the first study facility [ASFacility01], stopped issuing LLINs to ANC registrants for a couple of months during the period of observation. A health provider explained that they had reached the minimum stock, so they could not afford to give out the few that were available to new ANC registrants.

ANC managers in ASFacility02 and ASFacility03 reported that arranging transport services to convey LLINs from the DHD was a challenge. The ANC unit manager in AS Facility 02 said she was reluctant to use her own money to hire transport to convey LLINs from the DHD, because she will not be reimbursed by the accounts department, for lack of receipts:

*“The bed nets are always there [at the DHD], but the only problem is conveying the bed nets from the district health directorate to the ANC block, because of transportation problem. We are even ‘chasing’ the accountant to give us money to hire tricycle*-*motor to convey the bed nets from the district health directorate to the hospital [ASFacility02 is a twenty minute walk from the DHD, so the use of motor*-*tricycles is ideal, however motor*-*tricycles do not issue receipts for their services]. If we use our own money for the transport fare, the accountant will tell us to provide a receipt before our money will be paid, because every transaction has to be documented. So that is the main reason why we haven’t gotten bed nets for the past one week.”* (ASFacility02, IDI, ANC Manager).

A midwife and the procurement officer in Facility 03 explained that there was no transport, to convey LLINs from the DHD to the facility. However, during a visit to the facility at a later date, it was observed that LLINs had been brought to the Reproductive and Child Health Unit (RCH), which was a 3 min walk from the ANC unit. Yet LLINs were not being issued to ANC registrants. The excepts below is an account of the observation:

*“When I got there [ANC unit of Facility03], I saw two new registrants, so it prompted me to ask the in*-*charge [ANC manager] about the nets. She told me they had had new consignment. I waited patiently to see them give nets to the new registrants. Surprisingly enough no nets were issued to them, when they were leaving the facility. I reminded the in*-*charge of the nets for the new registrants. She told me the nets were not ready yet and they were going for them at the RCH unit. I headed straight to the RCH to inquire about the nets. The RCH unit manager said the consignment was actually meant for their department alone, but sometimes when the ANC runs short of nets, they give them part of their consignment.”* (ASFacility03, Observation notes, 03/09/2018).

The RCH manager further revealed that because Facility 03’s vehicle had broken down, she used her own money to hire a vehicle to convey the LLINs from the DHD to the facility.

In AS Facility 04, which was about four kilometres from the DHD, LLINs were in stock throughout the 1 year period that the facility was observed, so all registrants were issued LLINs. A health provider explained that they were in constant touch with the DHD, to ensure that they never run out of stock and the facility usually provided a vehicle to transport the LLINs from the DHD. She added that the ANC unit borrowed LLINs from the MCH unit, whenever they were out of stock (ASFacility04, IDI, Health Worker).

The narrations suggest that the frequent stock outs in the three study facilities in the Ashanti region was more of an artificial shortage, which was largely due to the lack of interest of the ANC managers to ensure that LLINs were available.

### Socio-cultural context-sensitive health information at ANC

Providing ANC registrants with comprehensive information that suited the socio-cultural context of registrants was crucial to LLIN use. All health workers interviewed in the eight facilities reported that they usually provided comprehensive information on LLINs to ANC registrants before issuing LLINs as follows: how to treat the LLIN before use, such as drying it in the shade for 24 h to prevent itchy body and eyes resulting from the chemicals used to treat it; relevance of LLINs in fighting malaria in pregnancy; and pregnant women needed to wear long-sleeved dresses, to prevent mosquito bites, if they had to stay outside for long periods during the evening.

Sometimes the interactions between health workers and ANC registrants who were reluctant to receive LLINs was more of a dialogue, where health workers had to provide suggestions to allay registrants’ fears and misconceptions about LLINs emitting heat and itching of the body and eyes. Health workers told them of the benefits to mother and the fetus, which appeared more appealing to them. They were also advised to use a fan to overcome the heat. Health workers also knew that some of the women used the LLINs for the wrong purposes, which also informed health workers on the kind of information to provide recipients:

“*When the registrant [pregnant women who register for ANC in a health facility] comes for the first time, we give you the bed net. We learnt that some of them use the bed net to fence their gardens, so now we are really doing education about the need to sleep under bed nets, because when you are pregnant and you get malaria, it is a very serious business.”* (ASFacility03, Interview with 2 Health Managers)

*“Because of the way they [pregnant women] are, when they come [to ANC], sometimes we have to plead with them not to go and use the net in their gardens. Some use the net to fence their gardens, because they say animals are destroying their crops. Because they complain of feeling hot in the net and out of ignorance, they use the net for fencing gardens”* (ASFacility01, IDI, Midwife)

It was observed that three out of six ANC registrants who were offered LLINs were not given comprehensive information, contrary to health managers and workers’ claims. On the other hand, pregnant women who received information on LLINs from health workers, were more likely to use one. Some testified that they were not using LLINs or were inconsistent users until they were issued LLINs at the ANC and asked by health workers to use them. Others stated that they were influenced to use LLINs through advertisements on LLINs on the radio/TV, encouragement from health workers and national leaders.

*“Before the pregnancy, I used it [LLINs] sometimes, but since I became pregnant, I was told at the hospital to use it always. So I sleep under it always.”* (VRCommunity04, IDI, Pregnant woman10).

*“He [former Ghanaian president Kufuor] spoke on radio concerning how we should sleep under bed net, so that mosquitoes will not bite us.”* (VRCommunity01, IDI, Pregnant woman 01)

“*They advertised it [LLIN] every day on TV and when we went to the hospital they told us [to use LLINs].”* (ASCommunity02, IDI, Pregnant woman 03)

### Social networks: influence of husbands, family interactions and friends

Household power dynamics between husbands and wives influenced women’s use of LLINs. Women who shared a room with their husbands had no power to determine LLIN use. When a husband supported the wife to hang the LLIN, encouraged her or insisted that she used it, she was likely to use one.

*“My husband makes sure I sleep under the bed net.”* (VRCommunity04, IDI, Pregnant woman 05)

*“My husband makes sure the children and I sleep under the bed net.”* (VRCommunity01, IDI, Pregnant Woman10)

*“…he [husband] scolds me on my refusal to sleep under bed net. When the scolds become too much, I try my best to use it.”* (VRFacility01, Conversation with a pregnant woman, 23/08/2018)

Husbands who were not keen in using LLINs, overruled their wives’ decision to use them. Women complied with their husband’s decision, as they were afraid to oppose them, even when they had knowledge of the effects of malaria in pregnancy and knew that their health was at risk.

*“Even if the pregnant women like to sleep under the net and the husbands don’t, they may have to compromise, because they think the husbands may use that as an excuse to sleep outside. The husbands often come to complain that they can’t sleep under the net with their wives.”* (ASFacility03, IDI, Health Worker 04)

In one of the facilities in the Volta Region, miscarriages in pregnancy was common and most of the women who had miscarriages in their previous pregnancies reported that they did not use LLINs during those pregnancies. Some of them reported that despite owning LLINs and receiving advice from health workers to use them, they were not using them, because of their husbands’ refusal, as the following quote from a woman who suffered miscarriage in her previous pregnancy suggests:

*“It’s my husband, he doesn’t like it [LLIN]. When I fix it on the bed he says he feels uncomfortable sleeping under. When I suggest he sleeps on the floor, he tells me to rather sleep there, whilst he and the children use the bed.”* (VRFacility03, conversation with an ANC attendant, 05/09/2018)

Pregnant women who lived in households that had several members such as mothers, mothers in-law and other family members using LLINs, were influenced positively to use them. Additionally, when other members of the household encouraged pregnant women to use LLINs, they did.

*“When I was coming… to this place I did not come with a bed net, but here everybody uses bed net and my mother in*-*law gave me one, before they gave me one at ANC.”* (VRCommunity01, IDI, Pregnant woman 03)

Interviewer*: “So, what would people in your household say if you do not sleep under a bed net?”*

Respondent: *“They will say that it is not good for me not to be sleeping under a bed net.”* (VRCommunity04, IDI, Pregnant woman 05)

Respondent: *“They will say I will fall sick, if I fail to sleep under a bed net. ‘And when you fall sick, there is no money to treat you.’*” (VRCommunity01, IDI, Pregnant woman 01)

Pregnant women who lived in households where LLINs were not being used, were most often not using them. A respondent who shared a room with her husband and child, owned two LLINs, one from the ANC and the other from the mass distribution campaign. Her household members were fourteen and received seven LLINs, yet none of them slept under an LLIN:

“*I have not done anything to it [bed net]. I have not even slept in one before….It’s my mum who used to have one, but I think it’s torn, so they haven’t hanged it*.” (ASCommunity01, IDI, Pregnant woman 01).

However, the household had used two new LLINs to fence a nursery of cocoa seedlings.

About half of respondents who complained of heat and itching from LLINs had never used them, though they owned LLINs and they only learnt about such reactions from friends, relatives and acquaintances.

Interviewer: *“I would like to know why you have 2 bed nets yet you don’t sleep under any!”*

Respondent: *“Not really anything. Just that my friends say they feel hot when they sleep under it [LLIN] that’s why I don’t use it.”* (ASFacility01, IDI, Pregnant woman 01)

Most of them were not able to give comprehensive account on the relevance of LLIN use and the negative effects of malaria in pregnancy.

Economic activities such as staying out late to ply their trade, to carry out household chores and to converse with friends and relatives exposed women to mosquito bites for several hours, before they went to bed. It was observed that some of the case studies who were visited at night stayed out for long hours, some wore long sleeved-dresses, as recommended by health workers, but others did not. A health worker expressed her frustration:

“*They use it [LLIN]. The only thing that I have seen as the mistake is that, most of the women spend too much time outside in the evening and get bitten by lots of mosquitoes before they get into their bed nets to sleep. We talk to them about that, but they say the heat in the room is too much and it is uncomfortable to go to bed that early*.” (VRFacility02, IDI, Health worker 03)

Also, some pregnant women stayed far off from their work places, so they had to share accommodation with others at their work places, where they could not use LLINs. Such women only used LLINs on their off days, when they returned to their homes.

### Individual factors

Individual factors such as building a habit of LLIN use, having children and knowledge on the effect of malaria in pregnancy encouraged women to use LLINs. Women who used LLINs prior to their current pregnancy were consistent LLIN users, both in the warm and cold seasons. Most of such women had received an LLIN in a previous pregnancy and were using it when not pregnant prior to receiving the new LLIN. Such women had acquired knowledge on the relevance of using LLINs as a result of having had more encounters with health care providers, due to having experienced multiple pregnancies. Such women shared their LLINs with their young children, which suggests that the motivation for using LLINs was to protect their young children, themselves and the unborn child:

“*The mosquito net that I am using was given to me after I gave birth to my child, who is now three years old. I have been using the mosquito net ever since …*” (ASFacility04, Conversation with an ANC attendant, 14/08/2018)

Another factor that promoted consistent use of LLINs among pregnant women was having knowledge on malaria and its negative effects on pregnancy. Women who reported that they were consistent users of LLINs and had used one the previous night were able to explain that malaria was a deadly diseases, it could kill the pregnant woman and can cause miscarriages. They also knew the signs and symptoms of malaria in pregnancy. Others simply said no one encouraged them to use LLINs, it was based on personal knowledge and some said they hated mosquitoes.

“*Oh, there are mosquitoes around, so I know it myself that I have to sleep under mosquito net, else I can contract malaria, if mosquitoes should bite me*.” (VRCommunity03, IDI, Pregnant woman 02)

Common reasons that respondents gave for not using LLINs were that they felt a burning sensation and some said their bodies itched when they used them. A respondent shared her experience:

“*I have it but I don’t like sleeping in it. If I sleep in it my body itches*.” (ASCommunity04, IDI, Pregnant woman05). A few said they had a natural fear when they used one and others said they simply did not enjoy using it “*I do not enjoy sleeping under a bed net*.” (VRCommunity04, IDI, Pregnant woman 03).

Other reasons for non-use of LLINs included the use of alternatives such as coils, spray, herbs, grass and burning of orange peels, which are perceived to be potent in wading off mosquitoes. Some believed that there were no mosquitoes in their environment, while others said they sprayed their rooms, so there were no mosquitoes, thus they did not need to use LLINs. Others also believed that once they had taken sulfadoxine-pyrimethamine (SP), there was no need to use LLINs.

*“The midwife told me the drug [SP] will protect me from malaria and that is the main reason I don’t even sleep in the net. Because if I have been given a drug [SP] that protects me from malaria, why then should I sleep in the net.”* (ASFacility02, Conversation with a 19 year old ANC attendant, 30/08/2018)

### Environment, type of housing, improper hanging of the LLINs

Pregnant women used LLINs more frequently during the rainy season, but refused to use them during the warm season, when temperatures were over 30 Degrees Celsius. A health manager lamented that despite the information on LLINs that pregnant women receive at ANC, they still complain that there is heat during the warm season. So they sleep naked without hanging LLINs, which exposes them to mosquito bites. (VR Facility 01, IDI, ANC Manager)

Visits to the homes of some of the case studies revealed that some of the houses, had very tiny windows and poor ventilation, so the rooms were very warm and very uncomfortable to sleep in during the warm season. Additionally, some women could not afford to buy a fan and a few could not afford to have electricity in their homes, so they could not enjoy the luxury of using fans or air conditioners.

Some of the case studies that we visited at home could not afford beds, so they slept on mattresses on the bare floor. Yet they hanged LLINs and some even owned a second LLIN. Some respondents used the LLINs because they could not afford mosquito spray or they reacted to the use of mosquito coil.

“…*some people use mosquito coil. With the mosquito coil I don’t use it because whenever I use it I get catarrh. Some of them buy mosquito spray to spray their rooms…I don’t have money to buy it, so I have to sleep in the net*.” (AS Community 04, IDI, Pregnant woman 02)

The study observed that some of the users did not hang the LLINs well on the bed, which rendered them less protective. Some LLINs were not large enough to cover the entire bed and could not be tacked tightly into the corners of the bed, consequently the users were exposed to mosquito bites and developing malaria. For instance a study participant, who was 2 months pregnant and consistently used an LLIN came down with malaria, because her LLIN did not cover the entire bed, so she was exposed to mosquitoes.

Interactions with health workers indicated that previously health workers were assigned to hang the LLINs in homes for the women. However, due to the shortage of health staff the practice was stopped.

## Discussion

This study has demonstrated that an interacting network of health facility arrangements, health worker provision of appropriate contextual information on LLIN use, contextual factors such as socio-cultural, economic, environmental and individual factors influenced LLIN use among pregnant women.

Four facilities in the Volta region and one in the Ashanti region, were more effective in distributing LLINs compared to three facilities in the Ashanti region. The free distribution and the diverse strategies employed in distributing LLINs might have accounted for the effective distribution of LLINs in the five facilities. Similarly, Beiersmann et al. [22] found that free distribution of LLINs was quite effective in a randomized controlled LLIN intervention study in Burkina Faso. The finding confirms WHO’s recommendation that the most effective strategy to attain universal access to LLINs is through free distribution [3]. Nevertheless, this study contradicts studies that suggest that free LLIN distribution is not effective [23, 24]. The ineffectiveness of distributing LLINs to women reported in Mubyazi et al. [24] was because beneficiaries needed a voucher, paid a token and sometimes had to travel long distances to receive an LLIN.

More women in the Volta Region owned LLINs compared to the Ashanti Region, which is a reflection of how institutional gaps influenced access to LLINs. The lack of enthusiasm to regularly restock LLINs with excuses of lack of transport to convey LLINs and that a facility could not issue LLINs because it had experienced minimum stock, might stem from the fact that health care providers knew that pregnant women sometimes did not use them for the right purpose. A study in Zambia on the other hand, found that the reason for stock outs and limited distribution of LLINs was due to limited number of LLINs given to the district health administration and the administration’s inability to obtain funds to buy LLINs [25].

It appeared that there was poor coordination between the different LLINs distributing channels, which resulted in some women owning several LLINs, while others did not own any. Also verification could have been done to determine whether women who registered for ANC in some facilities were given LLINs, as some women lost out due to the policy of not giving LLINs to women who had registered for ANC at different facilities. Owning several LLINs contributed to their wrongful use. Similarly, studies in Ethiopia found that there was poor coordination between the different stakeholders involved in the distribution of LLINs, which resulted in some households receiving excess LLINs and thus misusing the spare ones [26]. Ernst et al. [27], Ordinioha [28], McLean et al. [29, 30] have reported on the misuse of LLINs in Kenya, Nigeria, Tanzania and Uganda, respectively.

The depth of information on LLINs provided by health workers played an important role in the use of LLINs, yet three in six pregnant women did not receive information, when they were given LLINs. Also health workers’ ability to negotiate and consistently remind women to use LLINs helped women to deal with the socio-cultural context that they used LLINs. Women who received information from health workers, the radio/TV and from public figures had positive attitudes towards LLINs and consistently used them. Family members, community members and friends, had a strong influence on pregnant women’s use of LLINs. Similarly, a study in Ethiopia found that pregnant women had a positive attitude towards malaria and LLINs use when they received adequate information [31]. The study also found that information from the TV/radio and health extension workers was more important to the women and influenced them to change positively compared to information from friends and neighbours [31]. An earlier study in Ghana similarly found mothers, sisters and husbands as the main influencers to pregnant women’s use of LLINs [32]. Also, Bowen’s [33] study in Cameroun reported that media campaigns and free distribution of LLINs promoted ownership, use and the development of the culture of LLIN use.

Some pregnant women believed that IPTp, LLINs, spray, mosquito coil, weeds and herbs could be used exclusively or they could alternate them. Such perceptions exposed some pregnant women to malaria infection. A study in Nigeria found that more than half of pregnant women used SP during their most recent pregnancy for the prevention of malaria, but only one in six consistently used LLINs at night [34].

The biggest problem in both regions appeared to be inconsistent use of LLINs. Most inconsistent users attributed it to ‘feeling hot’ and some of those who had never used a LLIN gave the same reason. Such a perception was mostly due to speculation, misconception, ignorance, no information received and ill advice received from relatives and friends. Similarly, studies in Nigeria and Tanzania revealed that ownership of LLINs did not necessarily translate into use. Factors that influenced use were knowledge that LLINs prevent malaria and not holding misconceptions about malaria prevention [35, 36].

Staying out late to ply their trade, to socialize with friends, supervise children and relatives exposed women to mosquito bites. This rendered the use of LLINs ineffective. A study attests that livelihoods that expose women to long hours of being outside such as fetching fuel wood at dawn and water at dusk, exposed them to mosquito bites and malaria infection [37].

Cold season promoted use of LLINs, while warm season discouraged use in all the five study districts. There was probably no difference in behaviors among women in the two regions, because the chosen districts in the two regions share similar climatic conditions of warm and cold seasons. Similar findings of using LLINs more in the cold compared to the warm season have been reported in studies in Northern Ghana and in the Tafea Province of Vanuatu [38, 39].

Cleaning one’s environment, the use of long sleeved dresses at night, using mosquito coils, sprays, burning herbs and grass were also practiced among some of the women in the twelve study communities. This suggests that pregnant women combined information provided by health workers, as well as those provided by community members. Atkinson et al. [38], equally observed that community, household and personal cleanliness were crucial protective measures against malaria in Vanuatu.

The culture of couples sharing sleeping space fostered gender power relations between husbands and wives, which influenced decisions on LLIN use. Where husbands encouraged or insisted on LLIN use, wives complied and where husbands opposed, wives did not use LLINs, even if they had comprehensive knowledge. More women in the Volta compared to the Ashanti region reported husbands’ role in deciding the use of LLINs. The reasons might be because the culture in the two districts in the Volta region is patriarchal, unlike that of the Ashanti Region, which is matriarchal. Also a good number of women in the three districts of the Ashanti region lived in their natal homes, whereas those in the Volta Region lived in their husbands’ homes or in their husbands’ family homes. Thus husbands in the Volta Region wielded more power in household decision-making than their counterparts in the Ashanti region. Similarly, studies in Nigeria found that about thirty percent of pregnant women were influenced to use LLINs by partners [40]. Other studies in sub-Saharan Africa attest to the role of male involvement in HIV [41], maternal and child health services [42], as well as in family planning decision making [43]. The finding compares with other studies in developing countries, which suggest that cultural norms influence intra-household gender relations. Women have little bargaining power, so they lose out in critical decisions that affect them and their children’s health in favour of men [44–48].

Socio-economic status had a mixed influence on use, women who could not afford electricity and the cost of fan could not benefit from the advice from the health workers to use fans. However women with low income, who could not afford mosquito spray, owned and used LLINs. The easy access can be attributed to the Ghanaian strategy of distributing free LLINs through multiple sources. Our finding corroborates with Mwandama, Gutman’s [14] study that reported that poverty was not a barrier to LLIN ownership and use, due to the free LLIN distribution policy in Malawi.

## Limitation of the study

This study was not able to conduct an in-depth exploration of the link between miscarriages and the use of LLINs, which is an area that we recommend further research. Focusing on five districts in two regions makes it difficult to generalize the study findings. However the study’s strength lies in the fact that it describes and explains how individual behaviors in the use of LLINs is connected to health system and a socio-cultural context, which could only be interrogated using a qualitative research methodology.

### Implication of findings and recommendations

The three strategies employed in the distribution of LLINs is commendable, for it ensured that no social category of pregnant women were excluded, as both rich and poor women enjoyed ownership and use of LLINs.

For effective LLIN distribution and use it is crucial that health facilities and workers become motivated in promoting the goal of preventing malaria in pregnancy. ANC unit managers need to improve coordination with the MCH and other relevant departments that support the restocking of LLINs. This will ensure that they have uninterrupted supply of LLINs for ANC registrants.

Provision of comprehensive information on LLINs that is relevant to the socio-cultural, economic and environmental context that women live in is crucial. It should be emphasized that malaria interventions are not exclusive, they complement each other and so they should be used concurrently.

An intervention that seeks to improve LLIN use among pregnant women in Ghana needs to take a holistic approach, where entire communities and households are targeted for maximum impact.

This study backs the recommendation by Balami et al. [40] that health education should be factored into post-abortion care and there should be improvement in social and spousal support. This study further recommends widening the network to include more radio/TVs adverts, mothers, mothers-in-law, peers and experienced pregnant women as change agents.

## Conclusion

This ethnographic study found that the introduction of LLINs to help prevent malaria and its negative consequences in pregnancy in Ghana in the last twenty years, has produced mixed results. Some women still experience malaria in pregnancy due to multiple level factors such as health system gaps, socio-cultural, economic, environmental and individual negative attitudes of some women that hinder LLIN use. Nevertheless, the study found that there were some positive outcomes as these multiple level factors sometimes worked in women’s favour-pregnant women who received social support, context-sensitive information from health workers and easy access to LLINs used them. The study thus recommends a holistic approach to intervention. However, the study could not adequately establish the link between failure to use LLINs and miscarriages in one study community. Therefore, it recommends an in-depth exploration to understand the link between none-use of LLINs and miscarriages in the community.

## Data Availability

The datasets used and/or analysed during the current study are available from the corresponding author’s institution on reasonable request.
